# Transcriptome and WGCNA Analyses Reveal Key Genes Regulating Anthocyanin Biosynthesis in Purple Sprout of Pak Choi (*Brassica rapa* L. ssp. *chinensis*)

**DOI:** 10.3390/ijms252111736

**Published:** 2024-10-31

**Authors:** Chaomin Xu, Hui Huang, Chen Tan, Liwei Gao, Shubei Wan, Bo Zhu, Daozong Chen, Bin Zhu

**Affiliations:** 1 School of Life Sciences, Guizhou Normal University, Guiyang 550025, China; 14785696659@163.com; 2 Ganzhou Key Laboratory of Greenhouse Vegetable, College of Life Sciences, Gannan Normal University, Ganzhou 341000, China; 19914680321@163.com (H.H.); tanchen2020@gnnu.edu.cn (C.T.); gaoliwei@gnnu.edu.cn (L.G.); wanshubei@gnnu.edu.cn (S.W.); nczb615@163.com (B.Z.)

**Keywords:** sprout, anthocyanin biosynthesis, RNA-seq, expression pattern, WGCNA

## Abstract

Chinese cabbage is rich in vitamins, fibre, and nutrients and is one of the primary vegetables consumed in autumn and winter in South Asia. ‘Purple pak choi’ sprouts are particularly rich in anthocyanins and are favoured by consumers. However, reports on the regulation of anthocyanin synthesis in purple pak choi sprouts do not exist. In this study, we examined the phenotypic development of purple pak choi sprouts after germination. The total anthocyanin content increased from 0.02 to 0.52 mg/g FW from days 0 to 6. RNA-seq data analysis revealed an increase in differentially expressed genes corresponding to the development of purple pak choi sprouts. Expression pattern analysis of genes associated with the anthocyanin biosynthesis pathway revealed a significant upregulation of structural genes during the purple phase, suggesting that the transcription factors *PAP2* and *MYBL2* may play crucial regulatory roles. *BraPAP2.A03*, *BraTT8.A09*, and *BraMYBL2.A07* exhibited strong interactions with key genes in the anthocyanin biosynthesis pathway, specifically *BraDFR.A09*. Furthermore, the expression of *BraPAP2.A03* aligned with the expression patterns of most anthocyanin biosynthesis-related genes, whereas those of *BraTT8.A09* and *BraMYBL2.A07* corresponded with the expression pattern of *BraDFR.A09*. These results provide valuable insights into regulatory mechanisms underlying anthocyanin synthesis in purple pak choi sprouts.

## 1. Introduction

*Brassica rapa* L. (AA, 2n = 20), which originated in northern China, is an important vegetable crop of the Brassicaceae family. This species primarily encompasses three subclasses: Chinese cabbage, non-heading Chinese cabbage, and turnip, and is widely cultivated across Asia [1]. Chinese cabbage exhibits a high cold resistance, thrives in colder climates, and is particularly suitable for cultivation during cold seasons. Its popularity is largely attributed to its ability to provide an abundance of vitamins, fibres, and mineral nutrients essential for the human diet [2,3,4]. Fresh leaves and roots of Chinese cabbage serve as both medicinal and edible vegetables and the nutritional and health benefits of its sprouts have thus attracted increased attention in recent years [5,6]. Chinese cabbage also includes several purple varieties, such as Zicaitai, purple turnip, purple non-heading Chinese cabbage, and purple Chinese cabbage. Vegetative organs, including the cotyledons and true leaves of purple pak choi (*B. rapa* L. ssp. *chinensis*), exhibit dark purple colouration because of the presence of anthocyanins. The development of sprouts has a significant economic value.

Anthocyanins are natural, water-soluble pigments and secondary metabolites of flavonoids. The synthesis and accumulation of anthocyanins attract pollinators, facilitate seed dispersal, and enhance plant tolerance to biotic and abiotic stress [7,8]. Vegetables and fruits rich in anthocyanins, which are beneficial for human health, are becoming increasingly popular among consumers [9]. The biosynthesis and regulatory mechanisms of anthocyanins have been studied extensively for various plant species. This biosynthesis involves three main steps: phenylpropanoid synthesis, flavonoid synthesis, and anthocyanin synthesis and modification [10,11,12]. The first step is the phenylpropanoid synthesis pathway, which primarily includes three catalytic enzymes: *PAL*, *C4H*, and *4CL* [10]. The second step, flavonoid synthesis, mainly includes the catalytic enzymes *CHS*, *CHI*, *F3H* (*F3′H*, *F3′5′H*), and *FLS*. Finally, the biosynthesis and modification pathways of anthocyanins involve *DFR*, *ANS* (*LDOX*, *ANR*), *UGT*, *GST*, and *AT* protease [13]. *FLS* and *DFR* catalyse the production of flavonols and anthocyanins by competing with flavonoids [14]. *ANS* and *ANR* are responsible for the biosynthesis of anthocyanins and proanthocyanidins, respectively [10]. Furthermore, the expression levels of these structural genes are regulated by the MBW complex, which is composed of MYB transcription factors, bHLH transcription factors, and WD40 proteins [10,14,15].

In recent years, extensive studies have been conducted on the localisation of anthocyanin regulatory genes, identification of components, and expression patterns of genes related to anthocyanin biosynthetic pathways in Chinese cabbage. He et al. [11] identified *BrMYB2*, an R2R3-MYB transcription factor located on chromosome A07, as the key gene controlling the dominant purple-head trait. In *B. rapa* L. subsp. *chinensis* var. *utilis* ‘Zicaitai’ and Chinese cabbage, the MYB transcription factor *BrMYBL2.1* is regarded as a negative regulator of anthocyanin biosynthesis [16,17]; whereas the bHLH transcription factor *BrEGL3.2* positively regulates this process [16]. In turnips, a single amino acid substitution in the R2R3 conserved domain of the *BrPAP1a* transcription factor inhibits anthocyanin synthesis [18]. He et al. [19] found that the transcription factors *BrMYB2* and *BrTT8* in purple-headed Chinese cabbage (*B. rapa* L. ssp. *pekinensis*) may activate the biosynthesis of anthocyanins, primarily cyanidin-3-sophoroside-5-glucoside. Zhao et al. [4] used transcriptome and metabolomic analyses to demonstrate that *UGT75C1*, which catalyses the formation of pelargonidin-3,5-O-diglucoside and cyanidin-3,5-O-diglucoside, might play a crucial role in the development of purple leaves in non-heading Chinese cabbage. Previously, we identified *BraANS.A3* as a key gene controlling the purple leaf colour of pak choi (*B. rapa* L. ssp. *chinensis*), suggesting that the insertion of two short fragments into the promoter region of green Chinese cabbage may disrupt its normal expression [20]. Notably, purple pak choi begins to synthesise and accumulate anthocyanins in substantial quantities in the cotyledon stage. However, the biosynthesis and regulatory mechanisms of anthocyanins during the cotyledon stage in Chinese cabbage have not been identified.

In this study, we investigated the phenotype of pak choi from 0 to 6 d after germination and analysed the total anthocyanin content. We observed that the total anthocyanin content of pak choi was significantly different after 1 day and peaked on the 6th day. Subsequently, we selected the cotyledons of pak choi germinated for 0, 1, 2, and 6 d for comparative transcriptome sequencing analysis. Our results indicated that the number of differentially expressed genes (DEGs) increased during the development of purple pak choi sprouts and that these DEGs were enriched in metabolic pathways. Weighted Gene Co-expression Network Analysis (WGCNA) revealed a strong interaction between *BraPAP2.A03*, *BraTT8.A09*, and *BraMYBL2.A07* and their target genes, *BraDFR.A09*. Furthermore, quantitative reverse transcription polymerase chain reaction (qRT-PCR) confirmed that the expression pattern of the positive regulator *BraPAP2.A03* was consistent with most anthocyanin biosynthetic genes (ABGs), whereas the expression pattern of the negative regulator *BraMYBL2.A07* aligned with those of *BraDFR.A09* and *BraTT8.A09*. Our study provides new insights into the transcriptional regulatory mechanisms of anthocyanins in purple pak choi sprouts as well as a foundation for their development and utilisation.

## 2. Results

### 2.1. Phenotypic and Total Anthocyanin Content Analysis of Purple Pak Choi Sprouts in Different Developmental Stages

**Figure 1:**
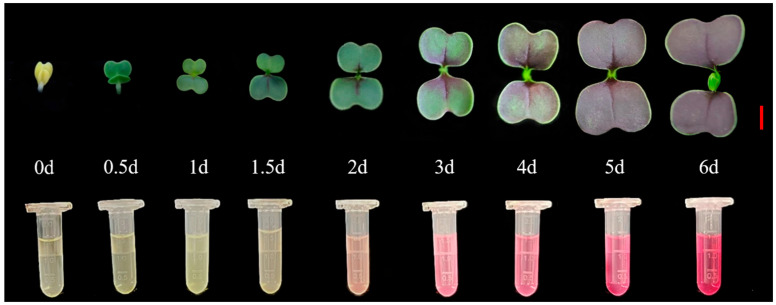
Phenotypes of purple pak choi sprouts in different developmental stages. Bar: 1 cm; d: day.

Purple pak choi sprouts are rich in anthocyanins that confer significant health benefits. In this study, we investigated the phenotypic development of purple pak choi sprouts from days 0 to 6 post-germination (Figure 1). On day 0, pak choi began germinating. Cotyledons had not yet expanded, appearing pale yellow because of the absence of synthesised chlorophyll. After 0.5 days of exposure to light, cotyledons synthesised a substantial amount of chlorophyll, resulting in green colouration, and exhibited semi-expansion. After 1 day of illumination, the cotyledons were almost fully expanded, with punctate purple spots observable at the junction of the cotyledons and hypocotyls near the growth point, indicating the initiation of anthocyanin synthesis and accumulation. After 1.5 days of illumination, banded purple patterns emerged on the cotyledons as anthocyanin synthesis and accumulation accelerated. After day 2, the cotyledons were flat, and the purple regions increased markedly. On day 3, almost half of each cotyledon was purple. After day 4, most of the cotyledons had turned purple. On day 5, the entire cotyledon surface was dark purple. Finally, on day 6, the purple colouration deepened further, and the growth point of the purple pak choi sprout began to develop its first true leaf.

**Figure 2:**
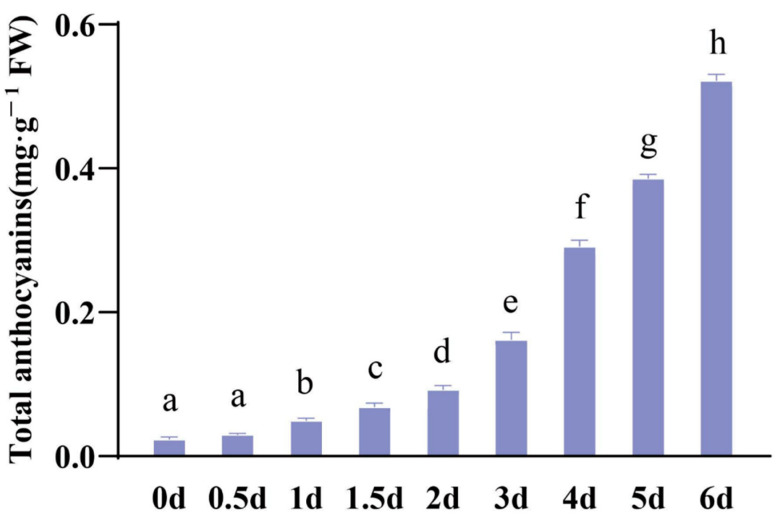
Analysis of the total anthocyanin content in purple pak choi sprouts in different developmental stages. Data are presented as the mean ± SD (n = 6). Different letters indicate significant differences (*p* < 0.05).

Furthermore, we extracted total anthocyanins from pak choi sprouts over nine periods ranging from 0 to 6 days. The colour of the total anthocyanin extract in the test tubes increased progressively over time. Similarly, we measured the total anthocyanin content of these samples using a microplate reader. It ranged from ~0.02 mg/g FW on day 0 to 0.52 mg/g FW after 6 days (Figure 2). The analysis of the total anthocyanin content revealed a significant difference after 1 d, that is, a marked increase in the total anthocyanin content. These results indicate that the synthesis and accumulation of total anthocyanins in pak choi sprouts commence at germination and increase significantly as the plants grow.

### 2.2. Analysis of DEGs in Different Developmental Stages of Pak Choi Sprouts

**Figure 3:**
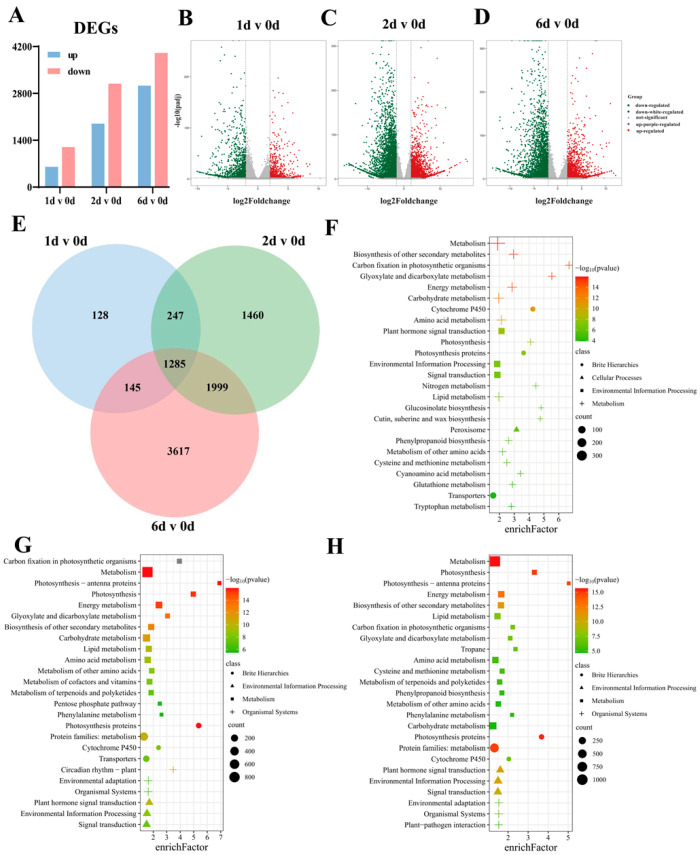
Identification and functional annotation of DEGs in different developmental stages of pak choi sprouts. (**A**) Differentially expressed genes (DEGs) analysis between different groups; (**B**) Volcano map of DEGs between 1 d and 0 d groups; (**C**) Volcano map of DEGs between 2 d and 0 d groups; (**D**) Volcano map of DEGs between 6 d and 0 d groups; (**E**) Common and specific DEGs between three groups; (**F**) KEGG pathway enrichment analysis of the DEGs between 1 d and 0 d groups; (**G**) KEGG pathway enrichment analysis of the DEGs between 2 d and 0 d groups; (**H**) KEGG pathway enrichment analysis of the DEGs between 6 d and 0 d groups. The screening conditions for DEGs were |log2Foldchange| ≥ 2 and *p*-value ≤ 0.01, enrichFactor: the number of DEGs in a term/the number of all DEGs.

To investigate the transcriptional regulatory mechanisms underlying anthocyanin synthesis and accumulation in purple pak choi sprouts as well as to identify key genes involved in anthocyanin biosynthesis, we analysed DEGs across the four developmental stages (Figure 1, Table S1). A total of 1805 DEGs were identified between the 1 d and 0 d groups, among which 608 were upregulated and 1197 were downregulated (Figure 3A,B). Between the 2 d and 0 d groups, we identified 4991 DEGs, comprising 1899 upregulated and 3092 downregulated genes (Figure 3A,C). In addition, a comparison of the 6 d and 0 d groups revealed 7042 DEGs, including 3033 upregulated and 4013 downregulated genes (Figure 3A,D). Notably, as purple pak choi sprouts developed, the number of DEGs increased significantly. We further analysed common and unique DEGs among the three comparison groups, revealing that 1285 DEGs were differentially expressed across these groups. Between the 1 d and 0 d, 2 d and 0 d, and 6 d and 0 d groups, 128, 1460, and 3617 unique DEGs were identified, respectively (Figure 3E). Interestingly, the trend observed in group-specific DEGs was consistent with the overall increase in DEGs. This increase in group-specific DEGs was identified as the primary factor contributing to an overall increase in the number of DEGs. Furthermore, we conducted a Kyoto Encyclopedia of Genes and Genomes (KEGG) enrichment analysis on the three groups of DEGs, which indicated that these DEGs were most enriched in metabolic pathways (Figure 3F–H).

In addition, we conducted a differential expression analysis between the 2 d and 1 d groups as well as between the 6 d and 2 d groups (Figure S1, Table S1). The results revealed 2116 DEGs between the 2d and 1d groups, with 865 upregulated and 1251 downregulated ones (Figure S1A,C). Furthermore, 4103 DEGs were identified between the 6 d and 2 d groups, comprising 1909 upregulated and 2194 downregulated genes (Figure S1A,D). Based on the combination of these results and those of differential expression analysis between the 1 d and 0 d groups, 202 DEGs were shared among the three groups, whereas the number of unique DEGs increased with the development of purple pak choi sprouts. KEGG enrichment analysis showed that the three groups of DEGs had the largest number of genes enriched in metabolic entry and the highest *p*-value. At the same time, multiple items, such as carbohydrate fixation in photosynthetic organisms, were also enriched (Figure S1F–H). These results were consistent with those of the phenotypic investigation and total anthocyanin content analysis. As purple pak choi sprouts develop, the synthetic pathways for anthocyanins and other metabolites are activated to regulate plant growth and development.

### 2.3. Expression Analysis of ABGs in Different Developmental Stages of Pak Choi Sprouts

**Figure 4:**
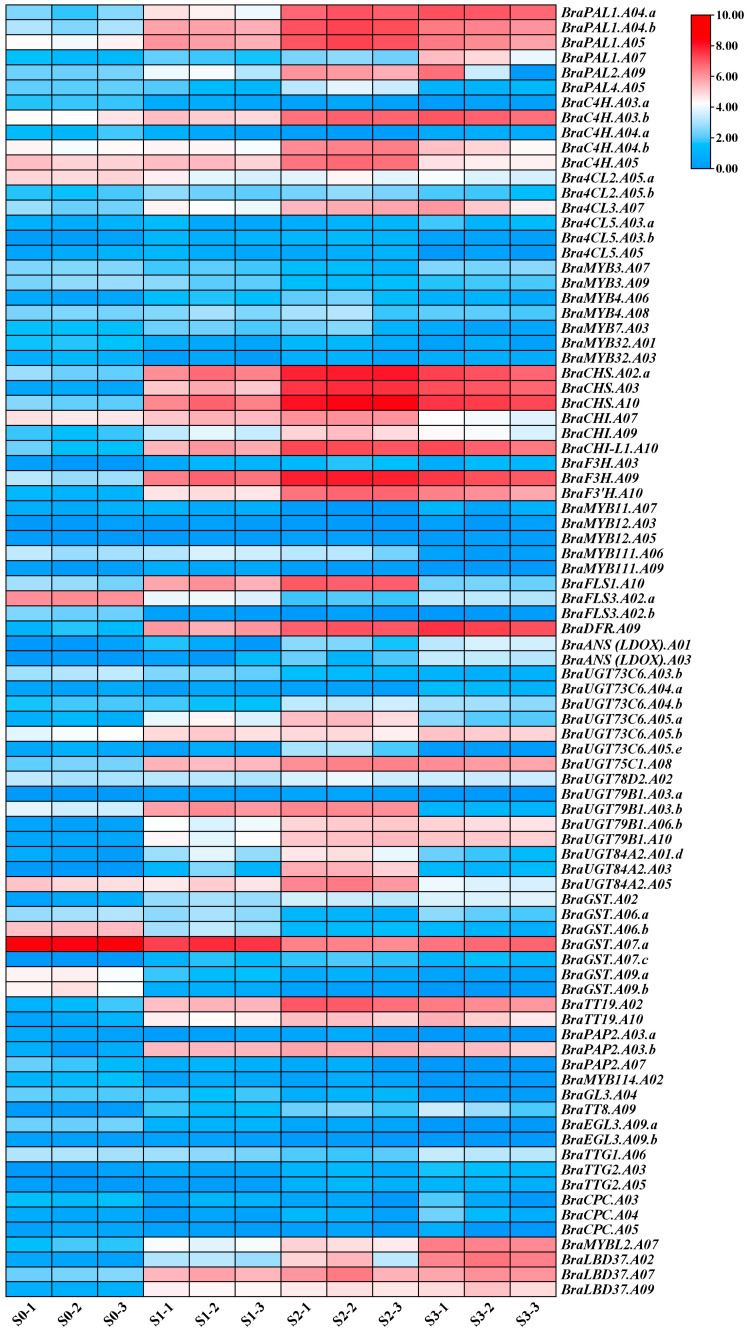
The expression patterns of anthocyanin-related genes in five tissues of purple pak choi sprouts were analysed using a heat map that represents the FPKM values of these genes. The colours on the heat map, ranging from blue to white and red, indicate the expression levels from high to low.

The results of our previous studies demonstrated that the synthesis and accumulation of anthocyanins are the primary reasons for the purple colouration of pak choi sprouts (Chen et al., 2023). KEGG analysis of DEGs revealed that these genes were significantly enriched in the metabolism and biosynthesis pathways of other secondary metabolites. To further investigate the expression of ABGs in various developmental stages of purple pak choi sprouts, we constructed an expression heat map of ABGs (Figure 4, Table S2). The results indicated that the expression levels of structural genes, such as *BraPAL1A04.b*, *BraC4HA03.b*, *Bra4CL3.A07*, *BraCHSA02.a*, *BraCHI-L1.A10*, *BraF3H.A09*, *BraF3′H.A10*, *BraDFR.A09*, and *BraTT19.A02*, were low on day 0 but significantly increased on days 1, 2, and 6. Furthermore, the expression patterns of the R2R3-MYB transcription factors *BraPAP2.A03.b* were consistent with those of the structural genes, agreeing with previous reports regarding the positive regulation of anthocyanin biosynthesis [11]. Notably, the expression of the R3-MYB transcription factor *BraMYBL2.A07* also significantly increased on days 1, 2, and 6; however, in contrast to the structural genes and *PAP2* transcription factors, its expression peaked on day 6. The results of previous studies suggested that the R3-MYB transcription factor *BrMYBL2.1* in Zicaitai and Chinese cabbage negatively regulates anthocyanin biosynthesis [16,17]. Therefore, as anthocyanin synthesis and accumulation increase, the expression of *BraMYBL2.A07* is upregulated in pak choi sprouts, potentially inhibiting further anthocyanin synthesis. In addition, the expression pattern of *BraLBD37.A07* was similar to that of *BraMYBL2.A07*, with expression levels gradually increasing as pak choi sprouts developed.

### 2.4. Co-Expression Network Analysis and Identification of Hub Genes for Anthocyanin Biosynthesis Regulation

**Figure 5:**
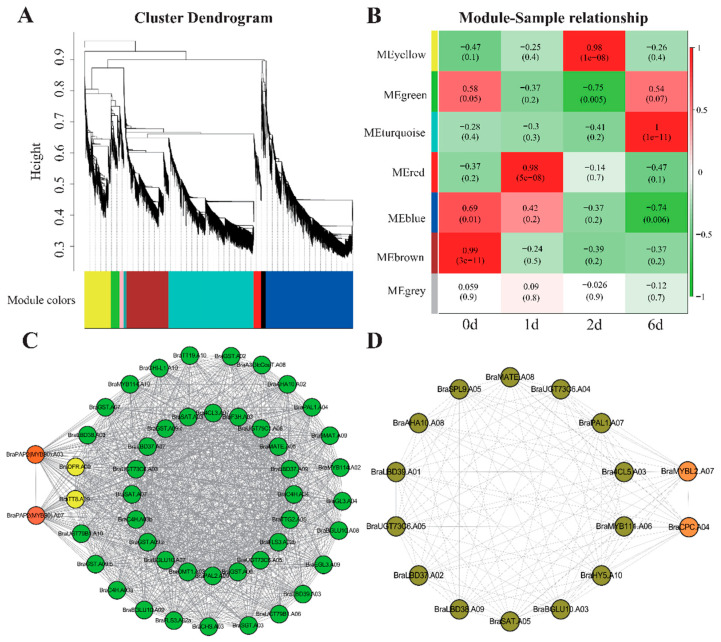
Weighted gene co-expression network analysis of DEGs in different developmental stages of pak choi sprouts. (**A**) Cluster dendrogram constructed by WGCNA showing seven co-expressed gene modules, the co-expression modules are depicted in different colours, while grey modules indicate no correlation between genes; (**B**) Module–sample relationship of seven modules, correlation analysis was performed between the co-expression modules of various genes in different developmental stages, the numbers above the heat map indicate the Person correlation coefficient (r) values; (**C**) Cytoscape representation of co-expressed anthocyanin metabolism-related genes in the MEbrown module; (**D**) Cytoscape representation of co-expressed anthocyanin metabolism-related genes in the MEturquoise module.

To further investigate the key genes regulating anthocyanin biosynthesis during the four developmental stages of purple pak choi sprouts, we used 8881 DEGs for weighted co-expression network analysis (Figure 5, Table S3). DEGs were categorised into nine branches based on their height (Figure 5A). We subsequently analysed the correlation heat maps among the seven main branches and observed that the correlation coefficient and threshold were the highest in the 6 d MEturquoise module and 0 d MEbrown module (Figure 5B). Furthermore, we examined the regulatory relationships among the 47 ABGs in the MEbrown module and identified key hub genes (highly connected genes). The analysis revealed that the key genes *BraPAP2.A03*, *BraPAP2.A07*, *BraTT8.A09*, and *BraDFR.A09*, which are involved in catalysing anthocyanin synthesis, were highly correlated (Figure 5C, Table S5). In addition, we assessed the regulatory relationships among the 16 ABGs in the MEturquoise module and identified hub genes. Notably, the key genes involved in anthocyanin synthesis, *BraMYBL2.A07* and *BraCPC.A04*, also displayed high expression correlations (Figure 5D, Table S5). The results of previous studies indicated that these two transcription factors function as negative regulators of anthocyanin biosynthesis (LaFountain AM, Yuan, 2021). These results suggest that the initiation of anthocyanin synthesis during the early stages of purple pak choi sprout development is driven by positive transcription regulators, such as *BraPAP2.A03*, *BraPAP2.A07*, and *BraTT8.A09*, which enhance the expression of *BraDFR.A09*, resulting in the synthesis and accumulation of significant amounts of anthocyanin. As the anthocyanin content increases, a negative feedback regulation mechanism is activated, leading to high expression of negative regulatory factors *BraMYBL2.A07* and *BraCPC.A04*, thereby inhibiting further anthocyanin synthesis.

### 2.5. Analysis of Expression Patterns of ABGs in Different Stages of Pak Choi Sprouts by qRT-PCR

**Figure 6:**
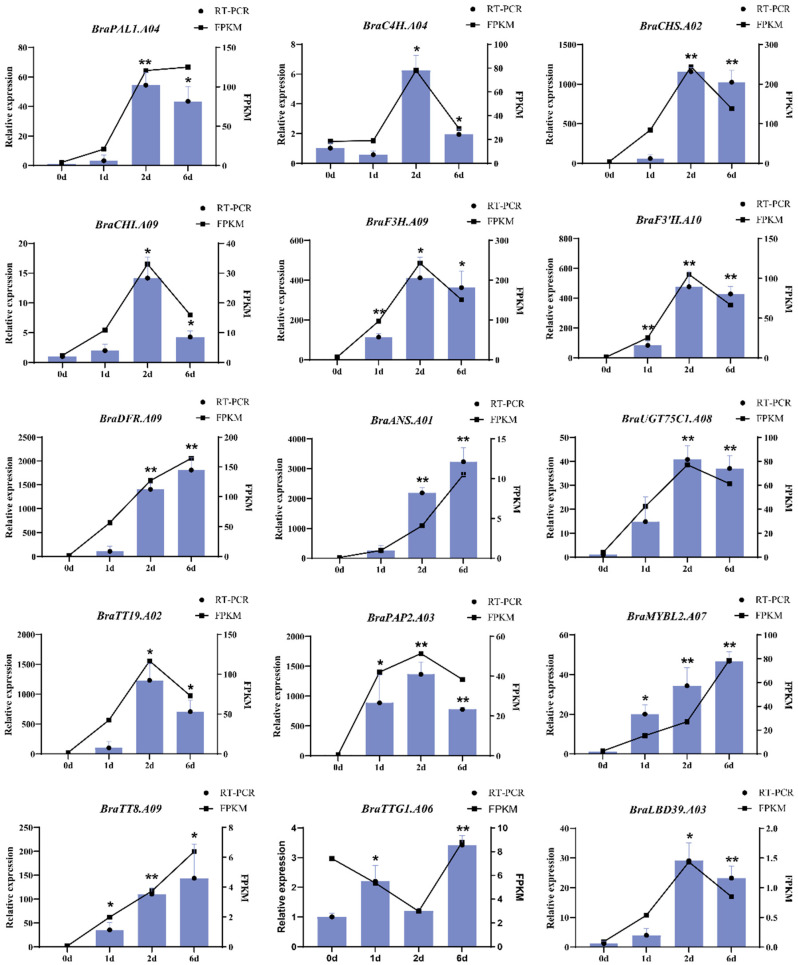
qRT-PCR analysis of expression patterns of anthocyanin biosynthesis-related genes in different developmental stages of pak choi sprouts. Both white and purple flower samples were utilised (with three biological replicates) for the analysis. Data points represent the mean value of three technical replicates in a representative biological experiment. Error bars indicate the standard deviation, student’s *t*-test, ** *p* < 0.01, * *p* < 0.05.

To further validate the results of the transcriptome and WGCNA analyses, 12 genes associated with the anthocyanin biosynthesis pathway were selected for qRT-PCR analysis. Alongside the expression level (FPKM) data from the transcriptome sequencing of these 15 genes, we generated Figure 6. The results indicated that the RNA-seq analysis for *BraC4H.A04*, *BraCHS.A02*, *BraCHI.A09*, *BraF3H.A09*, *BraF3′H.A10*, *BraDFR.A09*, *BraANS.A01*, *BraUGT75C1.A08*, *BraTT19.A02*, *BraPAP2.A03*, *BraTT8.A09*, *BraMYBL2.A07*, and *BraLBD39.A03* was consistent with the qRT-PCR verification results. Notably, the expression levels of *BraC4H.A04*, *BraCHS.A02*, *BraCHI.A09*, *BraF3H.A09*, *BraF3′H.A10*, *BraUGT75C1.A08*, *BraTT19.A02*, *BraPAP2.A03*, and *BraLBD39.A03* were significantly upregulated from days 0–2, peaked on day 2, and subsequently decreased until day 6. RNA-seq analysis of *BraPAL1.A04* on day 6 revealed a slight upregulation; however, the qRT-PCR results indicated that its expression level aligned with that of other structural genes, also showing a decline. Interestingly, the key enzymes *BraDFR.A09* and *BraANS.A01* and their crucial transcriptional regulators *BraTT8.A09* and *BraMYBL2.A07*, which facilitate the conversion of flavonoids to anthocyanins, were continuously upregulated during the development of pak choi sprouts.

## 3. Discussion

Chinese cabbage, which originated in China, has a long cultivation history in this region. It is favoured by consumers because of its rich nutritional value. Purple pak choi sprouts are particularly popular because of their significant market potential based on their high anthocyanin content. Although the regulatory mechanisms of anthocyanins in various subspecies of Chinese cabbage have been well-documented [11,16,17], the regulation of anthocyanin biosynthesis during the cotyledon stage of Chinese cabbage remains unexplored. This knowledge gap limits the development and utilisation of purple pak choi sprouts.

In this study, we investigated the phenotype of purple pak choi sprouts and measured their total anthocyanin content. Our results revealed that the purple phenotype deepened as the sprouts developed and the total anthocyanin content increased. In addition, we used RNA-seq data to analyse DEGs on days 0, 1, 2, and 6. These DEGs were predominantly enriched in metabolic pathways, which is consistent with our observations of the phenotype and total anthocyanin content. Notably, high expression of ABGs drives the synthesis and accumulation of anthocyanins, contributing to the purple phenotype of pak choi sprouts. We further examined the expression patterns of ABGs after 0, 1, 2, and 6 d and observed that the expression of structural genes was significantly increased in tissues with higher anthocyanin synthesis and accumulation, consistent with previous reports [16,21,22]. Furthermore, we used qRT-PCR to verify the expression of 12 ABGs and observed that the expression trends were consistent with the RNA-seq analysis results. Kim et al. [17] also used qRT-PCR to analyse the expression patterns of *BrCHS*, *BrCHI*, *BrF3H*, *BrF3′H*, *BrDFR*, *BrANS*, and *BrUFGT* in Chinese cabbage cotyledons. Similar to our results, the expression levels of these seven structural genes were significantly upregulated in the purple cotyledons. These results indicate that the expression of ABGs is crucial for driving the biosynthesis and accumulation of anthocyanins, which are key to the purple colouration of Chinese cabbage cotyledons, thereby providing a reference for further research.

Transcriptional regulation of anthocyanins in Chinese cabbage has been extensively documented. Transcription factors can be categorised as those that positively or negatively regulate this process [23,24]. Positive regulatory transcription factors primarily activate or upregulate the expression of structural genes involved in the anthocyanin metabolic pathway [11]. In contrast, negative regulatory transcription factors compete with positive regulatory factors for binding sites on structural genes, directly targeting positive regulatory transcription factors and inhibiting the expression of structural genes, thereby suppressing anthocyanin biosynthesis [25]. In this study, we employed WGCNA to identify the key genes that regulate anthocyanin synthesis and accumulation in purple pak choi sprouts. Interestingly, the highly correlated 6 d MEturquoise module and 0 d ME brown module of WGCNA appear inconsistent with the purple phenotype associated with anthocyanin accumulation. Further analysis revealed that the genes involved in the regulation of anthocyanin biosynthesis had not yet begun to express at 0d, and by 6 d, these genes had largely ceased expression. Consequently, through WGCNA analysis, we identified the modules with the highest correlation: the 6 d MEturquoise module and the 0 d ME brown module. Notably, the genes within the 0 d MEBrown module primarily play a positive role in regulating anthocyanin synthesis, whereas the 6 d MEturquoise module predominantly inhibits further anthocyanin synthesis. This distinction is particularly significant, prompting our subsequent regulatory network analysis to concentrate on the anthocyanin transcriptional regulation-related genes within these two modules. Then, the structural genes within the ABG network and the strong interactions between them were identified (Figure 6). We discerned transcription factors that regulate anthocyanin biosynthesis, particularly those that directly target *BraDFR.A09* and *BraANS.A01*. The transcription factors *BraPAP2.A03* and *BraTT8.A09,* which facilitate the conversion of flavonoids into anthocyanins, were identified as potential key regulators of anthocyanin synthesis and accumulation in purple pak choi sprouts by modulating the expression of *BraDFR.A09* and *BraANS.A01*. Results obtained by qRT-PCR analysis indicated that the expression trend of *BraTT8.A09* was consistent with those of *BraDFR.A09* and *BraANS.A01*, whereas *BraPAP2.A03* exhibited the highest expression level on day 2 and a decreased one on day 6. Notably, we observed that the expression pattern of *BraMYBL2.A07* mirrored those of *BraTT8.A09*, *BraDFR.A09*, and *BraANS.A01*. The results of previous studies on Chinese cabbage demonstrated that the homologous genes of *BraPAP2.A3.b*, that is, *BrMYB2* and *BrPAP1a* [11,18], can activate anthocyanin biosynthesis, whereas *BrMYBL2.1* is recognised as a negative regulator of anthocyanin biosynthesis in Zicaitai and Chinese cabbage [16,17]. Consequently, the role of *BraMYBL2.A07* in the regulation of anthocyanin synthesis in purple pak choi sprouts requires further investigation. In addition, during the 6-day period during which *BraMYBL2.A07* expression peaked, *BraTT8.A09* expression reached its highest level, whereas *BraPAP2.A3* expression was downregulated. Therefore, the relationship between *BraMYBL2.A07* and *BraPAP2.A03* requires further investigation.

## 4. Materials and Methods

### 4.1. Plant Materials

The experimental material selected for this study was *B. rapa* L. ssp. chinensis ‘Purple pak choi’. Initially, purple cabbage seeds were evenly sown in nutrient-rich soil. The illumination conditions of the incubator were set to a cycle of 16 h of light followed by 8 h of darkness. The temperature was maintained at 25 °C. The emergence of cotyledons breaking through the seed coat and soil was recorded as 0 days (0 d). The phenotypes on days 0, 0.5, 1, 1.5, 2, 3, 4, 5, and 6 were observed sequentially. Cotyledons were quickly placed in liquid nitrogen, frozen for 2 h, and then transferred to a −80 °C refrigerator for preservation. Six replicates were collected at each time point for the extraction and detection of the total anthocyanin content. Subsequently, based on the phenotype and total anthocyanin content test results, cotyledons from days 0, 1, 2, and 6 were selected, quickly frozen in liquid nitrogen for 2 h, and stored at −80 °C. Three replicates were performed at each time point to extract the total RNA for transcriptome sequencing and qRT-PCR verification.

### 4.2. Extraction and Analysis of the Total Anthocyanin Content

A 0.1 g sample of pak choi sprouts in various developmental stages (0, 0.5, 1, 1.5, 2, 3, 4, 5, and 6 d) was ground in liquid nitrogen and weighed. The powdered sample was then added to a 2 mL centrifuge tube. Subsequently, 1500 mL of extract, which consisted of a 50% aqueous methanol solution containing 0.1% hydrochloric acid, was added. The centrifuge tube was wrapped in foil and placed in an ice-water mixture for ultrasonic treatment for 30 min. Subsequently, the mixture was centrifuged at 4 °C for 10 min at a rotation speed of 12,000 rpm. The supernatant was carefully removed from light exposure and filtered through a 0.22 µm microporous membrane and then stored in a brown sample bottle in an ice bath for later use. A volume of 200 µL of the anthocyanin extract was added to a microplate, and the absorbance of each sample was measured at 530 nm. The total anthocyanin content (fresh weight) in each group of samples was calculated using the following equation: Q = (absorbance value A × 0.068 − 0.0037) × 1.5 × 10 (mg·g^−^¹).

### 4.3. Transcriptome Sequencing Data Analysis

Samples of 0, 1, 2, and 6 d pak choi sprouts were selected for transcriptome sequencing by BioYi Biotechnology Co., Ltd. (Wuhan, China). Database construction and sequencing methods were based on those described by [22]. The raw RNA-seq data obtained were mapped to the pak choi reference genome [26] using HISAT2 software (v2.1.0), ensuring that each read matched only one region. The htseq-count function of the HTSeq software package (v0.11.2) was used to count the number of reads aligned to each gene [27]. The DEseq2 package (http://www.bioconductor.org/packages/release/bioc/html/DESeq2.html accessed on 11 August 2024) was used to analyse DEGs. The expression level of each gene was calculated using StringTie software (v2.1.1) [26]. In addition, TBtools-II software (v2.096) was used to create a heat map of gene expression related to the anthocyanin synthesis pathway [22].

### 4.4. WGCNA Analysis

To further investigate the hub genes involved in regulating anthocyanin synthesis in the flowers of purple pak choi sprouts, we employed WGCNA to construct the interactions among DEGs across various stages of pak choi cotyledon development. Correlation analysis was conducted using the WGCNA software package (v4.3.1) [28] to assess the relationships among each co-expression module, phenotypic traits, and anthocyanin content, thereby identifying key hub genes that influence anthocyanin synthesis. The WGCNA analysis yielded a list of DEGs associated with anthocyanin content. From this list, we extracted genes related to the anthocyanin synthesis pathway and determined the regulatory relationship and weight values between these genes (Table S5). Subsequently, gene regulatory network data derived from the WGCNA analysis were imported into Cytoscape (v3.80) for visual representation [29]. For the detailed methodology, please refer to [30].

### 4.5. qRT-PCR Analysis

Purple pak choi sprout samples collected at 0, 1, 2, and 6 d were used to extract total RNA from which cDNA was synthesised through reverse transcription for qRT-PCR analysis. The specific methodology is as follows: First, the Eastep^®^ Super Total RNA Extraction Kit from Promega Biotech Co., Ltd. (Beijing, China) was utilised for total RNA extraction. Subsequently, a HiScript III 1st Strand cDNA Synthesis Kit (+gDNA wiper) from Vazyme Biotech Co., Ltd. (Nanjing, China) was used for cDNA synthesis by reverse transcription. qRT-PCR was conducted using SYBR qPCR Master Mix (Vazyme Biotech Co., Ltd., Nanjing, China). The reaction mixture comprised a total volume of 20.0 µL, consisting of 10.0 µL SYBR qPCR Master Mix, 0.5 µL of positive and negative primers each, 4.0 µL of cDNA template, and 5.0 µL of ddH_2_O. The amplification procedure included an initial denaturation step at 95 °C for 30 s, followed by 40 cycles of denaturation at 95 °C for 10 s, and annealing/extension at 60 °C for 30 s. This was followed by a final denaturation at 95 °C for 15 s, annealing at 60 °C for 60 s, and melting curve analysis at 95 °C for 15 s. The relative expression levels were calculated using the 2^−ΔΔCt^’ method. The fold change was determined based on the relative expression levels. Average values were visualised using Prism 9 (v9.5.1). Primer information for the qRT-PCR analysis is provided in Table S4. For methodological details, please refer to [31].

## 5. Conclusions

In this study, we observed purple pak choi sprouts over a period of 0 to 6 days and measured their total anthocyanin content. The results indicated that the purple colouration deepened progressively with increasing anthocyanin levels. Subsequently, we employed RNA-seq to analyse differentially expressed genes, and the expression patterns of genes associated with the anthocyanin synthesis pathway in purple pak choi sprouts at 0, 1, 2, and 6 days. By integrating WGCNA and qRT-PCR analysis, we identified key genes that regulate anthocyanin synthesis in purple pak choi sprouts, including *BraPAP2.A03.b*, *BraDFR.A09*, *BraANS.A01*, and *BraTT8.A09*. Additionally, we found that the negative regulator *BraMYBL2.A07* also plays a role in the transcriptional regulation of anthocyanin. Our findings provide valuable insights into the regulatory mechanisms governing anthocyanin synthesis in purple pak choi sprouts.

## Data Availability

All RNA-seq data in this study were uploaded to the NCBI database (https://www.ncbi.nlm.nih.gov/), with biological projects PRJNA1146938.

## Supplementary Materials

The supporting information can be downloaded at: https://www.mdpi.com/article/10.3390/ijms252111736/s1.

## List of Abbreviations

PAL: phenylalanine ammonia-lyase, C4H: cinnamate-4-hydroxylase, 4CL: 4-coumarate CoA ligase 4, CHS: chalcone synthase, CHI: chalcone isomerase, F3H: flavanone 3-hydroxylase, FLS: flavonol synthase, DFR: dihydroflavonol 4-reductase, ANS: anthocyanidin synthase, BAN: anthocyanidin oxidoreductase, UFT: UDP-flavonoid glucosyltransferase, GST: glutathione S-transferase, AT: acyltransferase, TTG1: transparent testa glabra 1, TT8: transparent testa 8, GL3: glabra 3, EGL3: enhancer of glabra 3, PAP1/2: production of anthocyanin pigment 1/2, MYB75/90/113/114: MYB domain protein 75/90/113/114, LBD37: lob domain-containing protein 37.
