# Supplementary material for: Transcriptome and WGCNA Analyses Reveal Key Genes Regulating Anthocyanin Biosynthesis in Purple Sprout of Pak Choi (Brassica rapa L. ssp. chinensis)
Source: Int J Mol Sci. 2024 Oct 31;25(21):11736. doi: 10.3390/ijms252111736 (PMC11546107; doi:10.3390/ijms252111736)
Supplement: Supplementary file 1 [file ijms-25-11736-s001.zip › Figure S1.pdf]

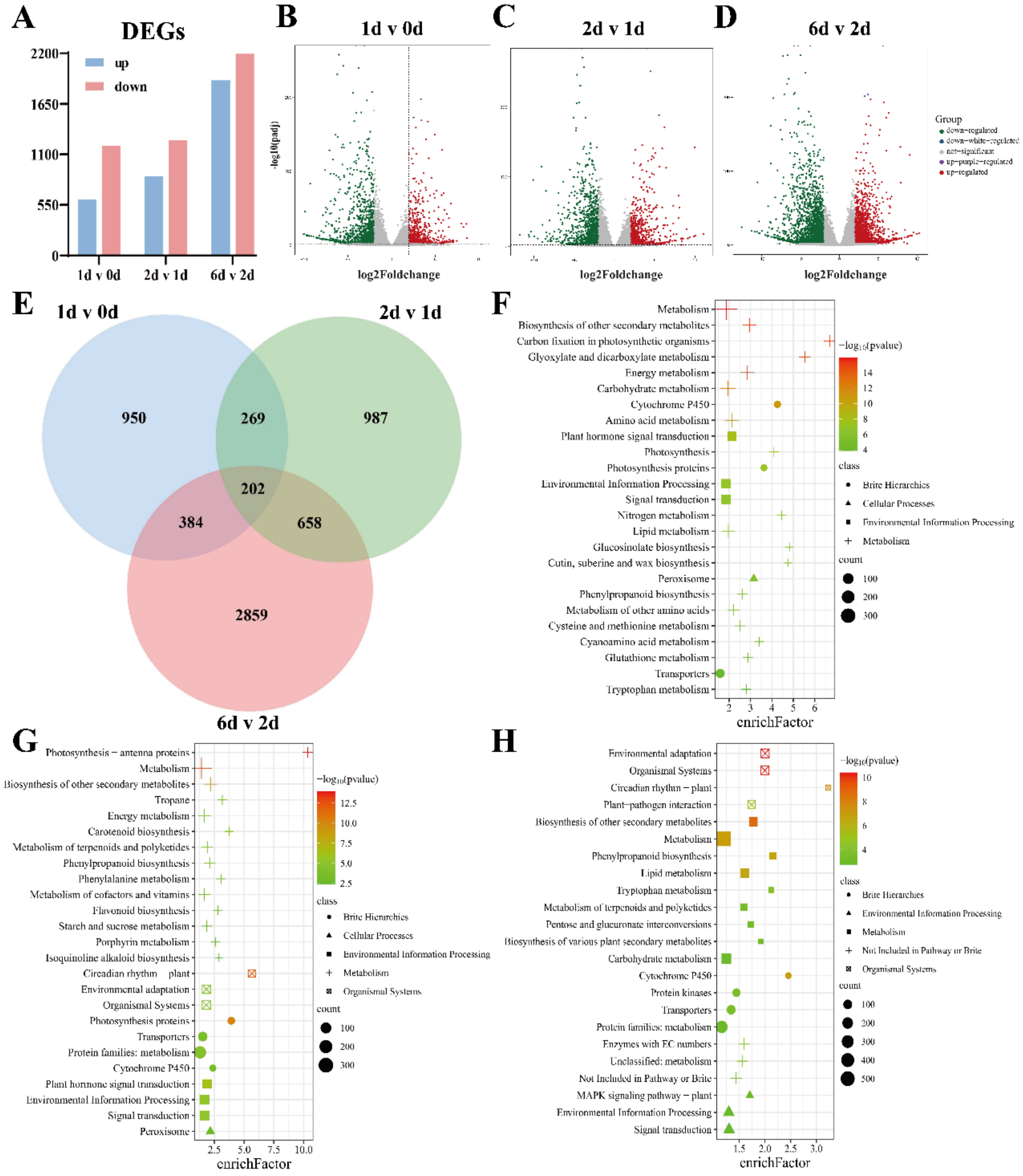

Figure S1 Identification and functional annotation of DEGs at four developmental stages of pak choi sprouts. A： DEGs between different groups; B: Volcano map of DEGs between 1d and 0d groups; C: Volcano map of DEGs between 2d and 1d groups; D: Volcano map of DEGs between 6d and 2d groups; E: Common and specific DEGs between groups; F: DEGs KEGG enrichment analysis between 1d and 0d groups; G: DEGs KEGG enrichment analysis between 2d and 1d groups; H: DEGs KEGG enrichment analysis between 6d and 2d groups.
